# Oridonin Triggers Chaperon-mediated Proteasomal Degradation of BCR-ABL in Leukemia

**DOI:** 10.1038/srep41525

**Published:** 2017-01-27

**Authors:** Huilin Huang, Hengyou Weng, Bowen Dong, Panpan Zhao, Hui Zhou, Lianghu Qu

**Affiliations:** 1 Key Laboratory of Gene Engineering of the Ministry of Education, State Key Laboratory for Biocontrol, Sun Yat-sen University, Guangzhou 510275, China

## Abstract

Inducing degradation of oncoproteins by small molecule compounds has the potential to avoid drug resistance and therefore deserves to be exploited for new therapies. Oridonin is a natural compound with promising antitumor efficacy that can trigger the degradation of oncoproteins; however, the direct cellular targets and underlying mechanisms remain unclear. Here we report that oridonin depletes BCR-ABL through chaperon-mediated proteasomal degradation in leukemia. Mechanistically, oridonin poses oxidative stress in cancer cells and directly binds to cysteines of HSF1, leading to the activation of this master regulator of the chaperone system. The resulting induction of HSP70 and ubiquitin proteins and the enhanced binding to CHIP E3 ligase hence target BCR-ABL for ubiquitin-proteasome degradation. Both wild-type and mutant forms of BCR-ABL can be efficiently degraded by oridonin, supporting its efficacy observed in cultured cells as well as mouse tumor xenograft assays with either imatinib-sensitive or -resistant cells. Collectively, our results identify a novel mechanism by which oridonin induces rapid degradation of BCR-ABL as well as a novel pharmaceutical activator of HSF1 that represents a promising treatment for leukemia.

Kinases have emerged as an important class of drug targets as accumulated evidence link aberrant kinase activity to oncogenesis^1^. Therefore, the development of kinase inhibitors such as small molecules and antibodies, has been a mainstay in seeking anticancer drugs in the past decade and leads to the discovery of several Food and Drug Administration–approved cancer therapies that benefit numbers of cancer patients^2^. Nonetheless, the facts that cancer cells acquire resistance to kinase inhibitors and that many oncogenic drivers are undruggable using this strategy prompt call for new strategies^2^,^3^.

The BCR-ABL gene, which results from a reciprocal translocation between chromosomes 9 and 22^4^, encodes a tyrosine kinase (TK) that contributes to the malignant phenotype of Philadelphia-positive (Ph^+^) leukemia cells and therefore has been an attractive target for leukemia treatment. Many famous tyrosine kinase inhibitors (TKIs), as represented by imatinib (STI-571, Gleevec), have been developed and approved for the first-line treatment of CML^5^. However, point mutations in the TK domain of BCR-ABL, along with Bcr-Abl gene amplification, result in relapse and resistance to TKIs therapy^6^,^7^,^8^. Furthermore, recent findings that BCR-ABL can function independent of its tyrosine kinase activity^9^,^10^ and that survival of CML stem cells is independent of BCR-ABL tyrosine kinase activity^11^ strongly encourage new strategies of targeting BCR-ABL (e.g., targeting BCR-ABL’s scaffolding ability or its DNA-binding ability, inducing degradation of BCR-ABL) to be used for improved therapeutic outcomes^2^,^3^,^12^.

Oridonin is a natural diterpenoid compound isolated from the traditional Chinese herb *Isodon rubescens* and shows promising anti-cancer activities, particularly for leukemia^13^,^14^,^15^. We have previously showed that oridonin targets c-Myc oncoprotein for degradation in an Fbw7-mediated manner^16^ and that by inhibiting c-Myc-regulated microRNAs, oridonin reverses chemoresistance in leukemia cells^17^. Here we report that oridonin inhibits the growth of Ph^+^ leukemia cells *in vitro* and *in vivo* by targeting BCR-ABL for degradation. Mechanistically, oridonin binds to and oxidizes cysteine-153 in HSF1, resulting in the nucleus relocalization and activation of this transcription factor. Activated HSF1 promotes the expression of HSP70 as well as the ubiquitin proteins UBB and UBC and subsequently triggers the HSP70/CHIP chaperone-E3 ligase complex-dependent proteolysis of BCR-ABL. Due to its distinct mechanism of action from that of TKIs, oridonin is not only efficacious in wildtype-BCR-ABL-expressing cells but also in cells with mutant BCR-ABL *in vitro* and *in vivo*. Our results reveal the role of oridonin as a small molecule proteostasis regulator that activates endogeneous HSF1 and support the potential clinical use of oridonin for BCR-ABL-expressing leukemia by inducing BCR-ABL degradation rather than inhibiting tyrosine kinase activity.

## Results

### Oridonin induces rapid depletion of the BCR-ABL fusion protein

**Figure 1:**
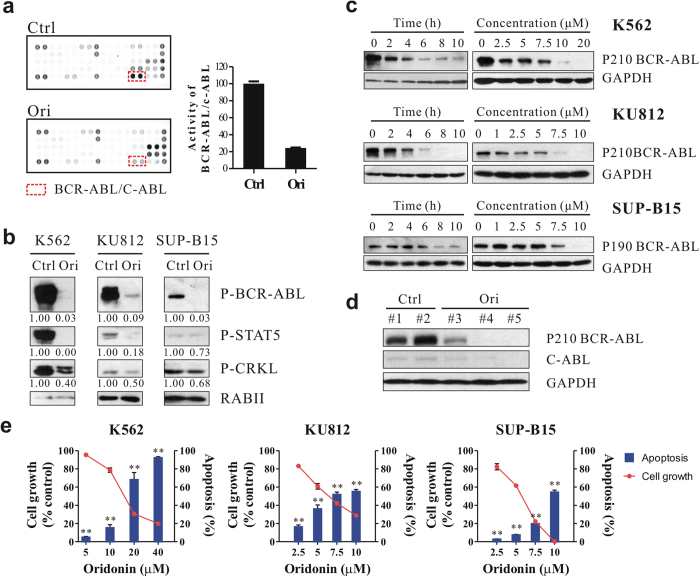
Oridonin depletes BCR-ABL and exhibits anti-proliferative and pro-apoptotic activities in Ph^+^ leukemia. (**a**) Protein array detecting the activity of tyrosine kinases. K562 cells were treated with DMSO (Ctrl) or oridonin (Ori, 20 μM) for 2 h and lysed for hybridization with the protein array. The quantification of BCR-ABL/C-ABL activity is shown in the right panel. (**b**) Immunoblots showing the activities of BCR-ABL and its downstream pathway in Ph^+^ leukemia cell lines after a 24-hour treatment with oridonin (20 μM for K562, 10 μM for KU812 and SUP-B15). RABII was used as a loading control. (**c**) Oridonin downregulates BCR-ABL protein levels in Ph^+^ cell lines in a time- and concentration-dependent manner as shown by immunoblotting. (**d**) Tumors from K562 xenograft mice treated with DMSO or oridonin (15 mg/kg) were subjected to immunoblotting. (**e**) Ph^+^ cells were treated with oridonin as indicated for 24 hours and cell growth and apoptosis were assessed by MTT assays and Annexin V/PI staining, respectively. **P < 0.01.

We have previously shown that oridonin exhibited potent anticancer activity towards Ph^+^ K562 CML cells *in vitro* and *in vivo*^16^. To deeply understand the underlying molecular mechanisms and to guide the use of oridonin in the clinic, we performed protein array assays to evaluate the effects of oridonin on the activities of tyrosine kinases. Noticeably, of the 28 tyrosine kinase proteins examined, the activity of BCR-ABL was substantially inhibited by oridonin in K562 cells after a 2-hour short-term treatment (Fig. 1a), suggesting a rapid and specific regulation. Western blots confirmed the reduction of phosphorylated BCR-ABL (p-BCR-ABL) and the inactivation of BCR-ABL downstream targets, STAT5 and CRKL, in multiple Ph^+^ cell lines, including K562, KU812 and SUP-B15 (Fig. 1b). Rather than just inhibiting the kinase activity of BCR-ABL, oridonin also reduced the total BCR-ABL protein in a time- and concentration-dependent manner in cultured cells (Fig. 1c). In xenograft tumor model, mice with oridonin treatment also showed decreased level of BCR-ABL protein in the tumor samples compared to those from vehicle-treated mice (Fig. 1d), indicating that oridonin could be a novel BCR-ABL-targeted agent.

Because Ph^+^ cells depend on BCR-ABL for their survival, we investigated the effects of oridonin on Ph^+^ cell lines. As expected, oridonin considerably reduced the percentage of viable cells and largely increased the percentage of cells undergoing apoptosis in a concentration-dependent manner (Fig. 1e), establishing the correlation between drug efficacy and the reduction of BCR-ABL.

### Oridonin promotes chaperone-assisted proteasomal degradation of BCR-ABL

**Figure 2:**
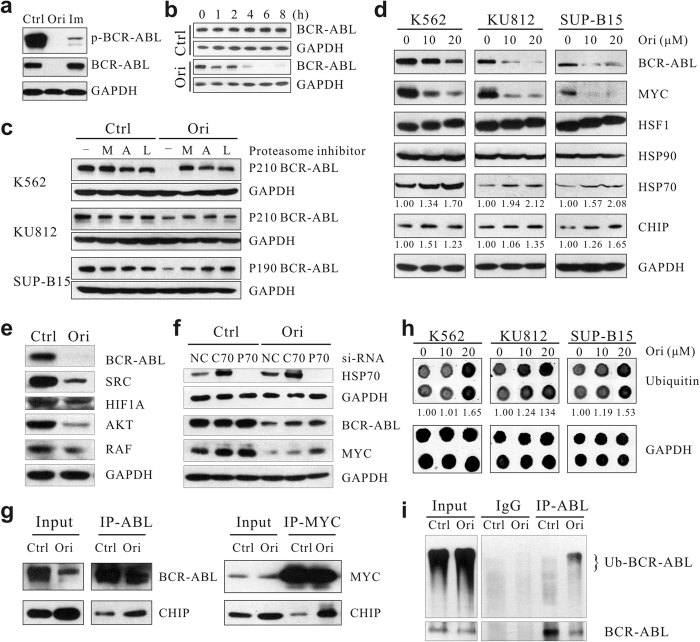
Oridonin triggers proteasomal degradation of BCR-ABL. (**a**) Distinct effects of oridonin and imatinib on BCR-ABL. Immunoblotting showing the total and phosphorylated Bcr-Abl protein levels in K562 cells after a 24 h drug treatment. Ctrl, DMSO; Ori, 20 μM oridonin; IM: 10 μM imatinib. (**b**) Cychloheximide chase assay showing the half-life of BCR-ABL protein in the absence or presence of 20 μM oridonin. (**c**) Proteasome inhibitors prevented oridonin-induced degradation of BCR-ABL. Cells were pretreated with proteasome inhibitors MG132 (M), ALLN (A) or lactacystin (L) for 2 hours, followed by treatment with oridonin (20 μM for 2 hours for K562; 10 μM for 2 hours for KU812; 10 μM for 6 hours for SUP-B15) and subsequent immunoblotting with an anti-c-ABL antibody. (**d**) Immunoblotting showing the effect of 2-hour of oridonin treatment on the expression of chaperons. (**e**) K562 cells were treated with 20 μM oridonin for 24 hours and subjected to immunoblotting analysis of downstream targets of BCR-ABL. (**f**) Silence of HSP70 by siRNA partially prevented oridonin-induced degradation of BCR-ABL and MYC proteins. K562 cells were transfected with negative control siRNA (NC), hsc70 siRNA (C70) and hsp70 siRNA (P70). Forty-eight hours later, cells were treated with 20 μM oridonin for 2 hours and subjected to immunoblotting. (**g**) K562 cells treated with DMSO or oridonin (20 μM) for 1 h were washed and lysed with IP lysis buffer and subjected to immunoprecipitation with an anti-c-Myc or anti-c-ABL antibody. The levels of CHIP protein in the IP products were examined by immunoblotting. (**h**) Protein dot blot showing the total levels of ubiquitin in Ph^+^ leukemia cells after a 2-hour treatment with oridonin. The relative expression of ubiquitin to GAPDH is quantified and shown. (**i**) K562 cells pretreated with MG-132 for 2 hours were then exposed to oridonin (20 μM) for 1 hour and lysed for IP.

Apparently Oridonin acts on BCR-ABL in a different way from imatinib as imatinib could only inhibit the tyrosine kinase activity but not the protein level of BCR-ABL (Fig. 2a and Supplementary Fig. S1a). In an attempt to understand the regulation of BCR-ABL by oridonin, we first performed RT-PCR and qRT-PCR and found that oridonin did not reduce the BCR-ABL mRNA levels (Supplementary Fig. S1b). Instead, CHX chase assays suggested that oridonin shortened the half-life of BCR-ABL protein, which was reported to be approximately 40 hours^18^, to less than 2 hours in K562 cells (Fig. 2b). Pre-incubation of Ph^+^ cells with proteasome inhibitors, including MG-132, ALLN and lactacystin, substantially prevented the depletion of BCR-ABL by oridonin (Fig. 2c), indicating that oridonin triggered proteasome-dependent degradation of BCR-ABL in these Ph^+^ cells.

Similar to MYC that has been demonstrated to undergo proteasomal degradation upon oridonin treatment^16^, MG-132-protected BCR-ABL proteins were largely enriched in the detergent (NP40)-insoluble fraction in the presence of oridonin (Supplementary Fig. S1c), which is consistent with the finding that proteins tagged with ubiquitin and yet to be degraded are insoluble in buffers containing only nonionic detergents such as NP40^17^,^19^,^20^. Moreover, the levels of HSP70 and its co-chaperone CHIP, the latter was previously shown as an E3 ligase of BCR-ABL and MYC^21^,^22^, were also elevated in the NP40-insoluble fraction (Supplementary Fig. S1c), suggesting that they were involved in oridonin-induced proteasomal degradation of BCR-ABL and MYC. Indeed, western blot analysis showed that oridonin upregulated HSP70 and CHIP protein levels in Ph^+^ cells (Fig. 2d) and decreased that of several chaperone substrates, including BCR-ABL, MYC, SRC, HIF1A, AKT and RAF (Fig. 2d,e), whereas the expression of HSP90 (Fig. 2d) and other cochaperones, such as Bag1, Bag2, p60 and p23 (data not shown), were not affected. Knock-down of HSP70, but not HSC70, partially blocked the reduction of BCR-ABL and MYC by oridonin in K562 cells (Fig. 2f), demonstrating its role in the turnover of these two substrate proteins upon oridonin treatment. It should be mentioned that siRNA against HSC70 induced the expression of HSP70 (Fig. 2f; Supplementary Fig. S1d), which was consistent with a previous report^23^. Furthermore, we found that oridonin treatment increased the amount of CHIP that bound to BCR-ABL and MYC (Fig. 2g) and increased the total levels of ubiquitin proteins (Fig. 2h). Similar to MYC^16^, the ubiquitination in BCR-ABL increased upon oridonin treatment (Fig. 2i). Taken together, these data indicates that oridonin enhances CHIP-mediated ubiquitination and degradation of BCR-ABL and MYC.

### Activation of HSF1 by oridonin for chaperone-mediated degradation of BCR-ABL

**Figure 3:**
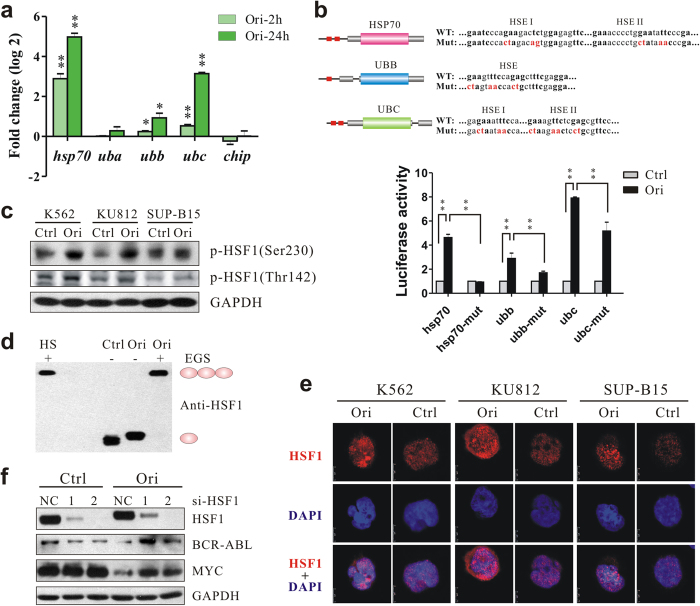
Oridonin activates HSF1 transcription factor and promotes the expression of HSP70 and ubiquitin. (**a**) qPCR showing changes of chaperons and ubiquitins in K562 cells after oridonin (20 μM) treatment. (**b**) HSE reporter gene assay. Upper, schematic diagrams showing the HSE elements (red boxes) in the promoter regions of *hsp70, ubb* and *ubc* genes. Mutations introduced for the luciferase reporter assays were shown in red. Lower, K562 cells were transfected with luciferase reporter plasmids and treated with DMSO or oridonin (10 μM). The activity of firefly luciferase was analyzed relative to that of renilla luciferase. *P < 0.05; **P < 0.01. (**c**) Immunoblotting showing the increase of phosphorylated HSF1 by oridonin (K562: 20 μM for 2 hours; KU812 and SUP-B15: 10 μM for 2 hours). (**d**) Detection of HSF1 trimer and monomer. Cell lysates from K562 cells treated with DMSO or oridonin (ori, 20 μM) for 2 hours, or heat shock (HS) at 42 °C for 30 minutes, were cross-linked with 1 mM EGS at room temperature for 20 minutes before subjected to immunoblotting. The position of HSF1 monomers and trimers are indicated on the right. (**e**) Immunofluorescence detecting HSF1 (Red) in Ph^+^ leukemia cells. Cells were treated with DMSO or oridonin (K562: 20 μM; KU812 and SUP-B15: 10 μM) for 24 hours, immuno-stained and analyzed by confocal microscopy. (**f**) Knockdown of HSF1 prevented oridonin-induced degradation of BCR-ABL and MYC. Forty-eight hours after transfection, K562 cells were treated with 20 μM oridonin for 2 hours and subjected to immunoblotting.

To gain a deeper insight into the mechanism of oridonin in chaperone-mediated degradation of BCR-ABL, we examined the mRNA levels of the above mentioned genes affected by oridonin. We found that oridonin increased the mRNA levels of *hsp70* and two stress-inducible polyubiquitin genes, *ubb* and *ubc*, in all three Ph^+^ cell lines (Fig. 3a; Supplementary Fig. S2). Noticeably, all these three genes possess validated or putative HSEs in their promoters (Fig. 3b), whereas genes without HSE in their promoters, including *uba* and *chip*, did not show consistent mRNA elevation (Fig. 3b; Supplementary Fig. S2). Furthermore, luciferase reporter assays showed that when placed upstream of the luciferase reporter gene, the HSEs of *hsp70, ubb* and *ubc* induced transcription of reporter gene in the presence of oridonin, and this was completely or partially abrogated when mutations were introduced into HSEs (Fig. 3b), suggesting the importance of HSEs in the regulation of these genes by oridonin.

Because HSF1 is the master regulator of transcription under protein-damaging conditions (e.g., heat shock) by binding to HSEs^24^, we examined the expression of the HSF1 protein under oridonin treatment. As shown in Fig. 2d, with the increase in oridonin concentration, the migration of HSF1 slowed down, resulting in the appearance of higher molecular-weight bands that might represent phosphorylated HSF1. Using phosphor-specific HSF1 antibodies, we demonstrated that oridonin indeed promoted phosphorylation of HSF1 at Ser-230 and Thr-142 (Fig. 3c), which contributes positively to the transcriptional activity of HSF1 according to previous reports^25^,^26^. When we cross-linked cell lysates with EGS, a shift of HSF1 from monomers to homotrimers was detected in oridonin-treated cells as well as in cells heat-shocked at 42 °C for 30 minutes (Fig. 3d). In addition, oridonin substantially promoted the formation of HSF1 granules in the nucleus (Fig. 3e).

All the above data imply the activation of HSF1 by oridonin in Ph^+^ cells. To directly evaluate the role of HSF1 in oridonin-induced degradation of BCR-ABL, we performed loss-of-function study using siRNAs. Short-term treatment of oridonin in K562 cells transfected with negative-control siRNA led to a considerable decrease in BCR-ABL and MYC protein levels, which could be largely prevented when HSF1 was silenced (Fig. 3f). Overall, these results provide evidence that oridonin activates HSF1 and its downstream chaperone-mediated proteolysis pathway, which in turn is required for the degradation of BCR-ABL and the anti-cancer efficacy of oridonin.

### Direct binding of oridonin oxidizes cysteine in HSF1

**Figure 4:**
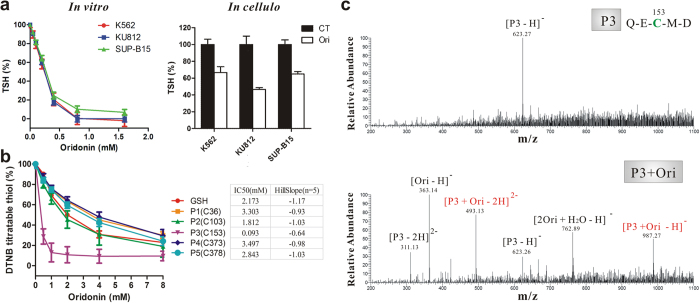
Direct binding of oridonin to HSF1. (**a**) Oridonin decreased the level of TSH *in vitro* and *in cellulo*. Left, cells lysates were incubated with oridonin at 37 °C for 10 minutes before DTNB reaction was performed to determine the TSH level. Right, cells were treated with oridonin for 2 hours and lysed for DTNB reaction. (**b**) Oridonin oxidized the thiols in GSH and synthesized peptides of HSF1. Peptides (2 mM) were incubated with oridonin at 37 °C for 10 minutes before DTNB was added. P1 to P5 represent peptides contain cysteines at position 36, 103, 153, 373 and 378 of HSF1, respectively. (**c**) ESI-MS showing the interaction between oridonin and HSF1. Peptide (P3) containing the cysteine 153 was incubated with DMSO (upper) or oridonin (lower) at a 1:1 molar ratio at room temperature for 10 minutes and loaded for ESI-MS.

It has been suggested that protein thiols are important for the activation of HSF1 under oxidative stress^27^,^28^,^29^ and that oridonin is potential to react with sulfhydryl (-SH, thiol) groups owing to its chemical structure^30^. To test the ability of oridonin to affect the redox state of cells, we examined the levels of total thiols (TSH). Our *in vitro* and *in cellulo* experiments both demonstrated that oridonin reacted with and decreased the levels of TSH (Fig. 4a). In addition, elevation of intracellular reactive oxygen species (ROS) was observed in Ph^+^ cells when treated with oridonin (Supplementary Fig. S3), consistent with previous reports using other cancer cell lines as models^14^. These results taken together imply that oridonin induces tremendous oxidative stress in Ph^+^ cells by simultaneously increasing ROS and decreasing TSH and support the notion that oridonin can react with thiol groups in cells.

Within the protein sequence of HSF1, there are five thiol-containing cysteines. To determine whether oridonin caused oxidative stress through interaction with HSF1, especially the thiol groups of its cysteine residues, we synthesized five peptides of HSF1, each containing one cysteine residue. When these peptides were pre-incubated with oridonin for 10 minutes at 37 °C, their activities to react with DTNB (a chemical commonly used for quantifying thiol groups) were decreased in an oridonin-concentration-dependent manner (Fig. 4b), indicating that oridonin oxidizes the thiol groups within these peptides. It is worth noting that oridonin has a greater potency to react with the C153-containin peptide (P3), with a much lower IC50 (0.093 mM) compared to those for the other peptides including GSH (Fig. 4b).

Furthermore, we used ESI-MS to assess the direct binding between oridonin and HSF1. In the absence of oridonin, the ESI/MS spectrum of the P3 peptide revealed only one major peak at m/z 623.27 representing the peptide itself (Fig. 4c). The addition of oridonin resulted in a transition from the P3 peak to the oridonin-binding peaks, as observed at m/z 493.13 and m/z 987.27 (Fig. 4c). The oridonin-binding peaks were also observed for GSH and the other five peptides of HSF1 (Supplementary Fig. S4a–f); however, only weak to medium signals were detected for these peptides, suggesting that these peptides have relatively low affinity for oridonin. Taken together, these data suggest that oridonin can directly bind to and oxidize cysteine residues, specifically and most likely cysteine-153, within HSF1.

### DTT counteracts HSF1 activation and attenuates inhibition of Ph^+^ cells by oridonin

**Figure 5:**
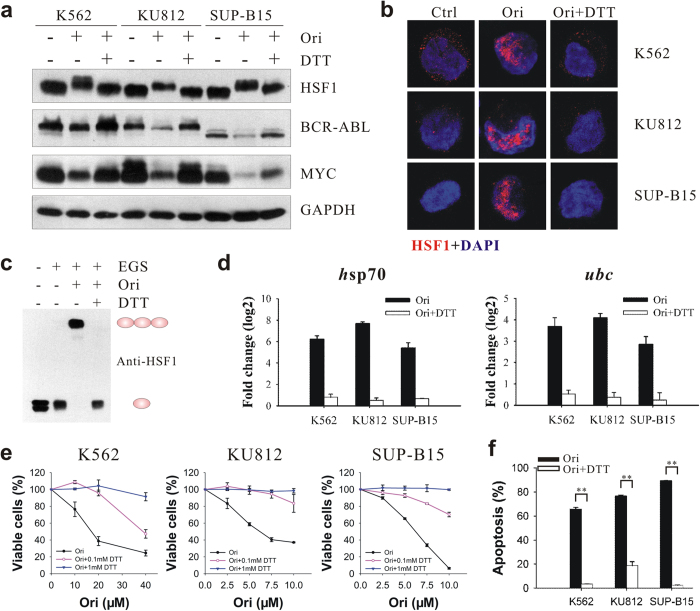
DTT prevents oridonin-induced activation of HSF1 and inhibitory effects in Ph^+^ leukemia cells. (**a**) Cells were pre-incubated with DTT (1 mM) for 2 hours and then treated with oridonin (K562: 20 μM for 2 hours; KU812 and SUP-B15: 10 μM for 4 hours) before subjected to immunoblotting. (**b**) Immunostaining showing the absence of HSF1 granules (red) in nucleus (blue) of oridonin-treated cells in the presence of DTT. (**c**) DTT prevents oridonin-induced HSF1 trimerization. K562 cells were treated with oridonin for 2 hours in the absence or presence of DTT. Lysates were cross-linked with EGS before subjected to immunoblotting. (**d**) DTT inhibits oridonin-induced transactivation of *hsp70* and *ubc*. Cells were treated with oridonin for 24 hours with or without DTT and subjected to qPCR analysis. (**e**) DTT abrogates the inhibition of cell growth by oridonin as assessed by MTT assays. (**f**) Oridonin-induced cell apoptosis in Ph^+^ leukemia cells was largely antagonized by DTT as determined by Annexin V/PI staining and flow cytometry.

To understand whether oxidation of HSF1 by oridonin leads to its activation, we used DTT, a thiol-reducing reagent, as co-treatment with oridonin. As expected, DTT counteracted the effect of oridonin on activating HSF1. First, the presence of 1 mM DTT completely prevented the phosphorylation of HSF1 and the degradation of BCR-ABL and MYC proteins by oridonin (Fig. 5a). Second, oridonin-induced nuclear localization and granules formation of HSF1 was completely blocked by DTT (Fig. 5b). Third, oridonin could not induce the formation of HSF1 homotrimer *in vitro* in the presence of DTT (Fig. 5c). Finally, DTT abrogated oridonin-induced increase in HSF1 transcriptional activity, as shown by the decrease of the *hsp70* and *ubc* mRNA levels to their original state in the three Ph^+^ cell lines by DTT (Fig. 5d). Consistent with its ability to antagonize oridonin-induced HSF1 activation, DTT attenuated the inhibitory effects of oridonin towards Ph^+^ cells, as shown by the restoration of cell viability and the protection from apoptosis by DTT in oridonin-treated cells (Fig. 5e,f).

### Oridonin triggers degradation of imatinib-resistant BCR-ABL and exhibits inhibitory effects *in vitro* and *in vivo*

**Figure 6:**
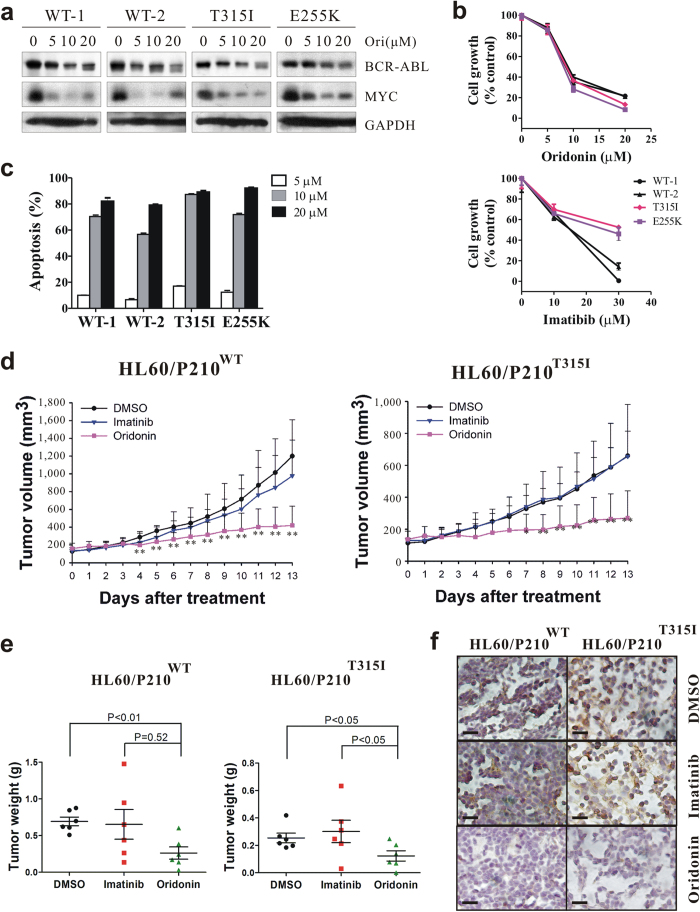
Oridonin degrades BCR-ABL and exerts anticancer effect in imatinib-sensitive and -resistant leukemia cells *in vitro* and *in vivo*. (**a**) Oridonin decreases wild-type and mutated BCR-ABL in HL60/p210 cells. HL60 subclones stably expressing wild-type (WT) or mutated (T315I, E255K) BCR-ABL were treated with oridonin for 6 hours and lysed for immunoblotting. (**b**) Oridonin inhibits cell growth in imatinib-sensitive and -resistant leukemia cells. MTT assays were performed after a 24-hour oridonin treatment or a 48-hour imatinib treatment in HL60 subclones. (**c**) Oridonin induces apoptosis in imatinib-sensitive and -resistant leukemia cells. Cells were treated with oridonin for 24 hours and subjected to Annexin V/PI staining and flow cytometry. (**d**,**e**) Oridonin inhibits tumor growth in HL60/p210wt and HL60/p210T315I xenograft-bearing nude mice as shown by tumor volumes (**d**) and tumor weights (**e**). (**f**) Immunohistochemistry staining of BCR-ABL on tumor sections. Bar = 50 μm. *P < 0.05; **P < 0.01.

The above results together reveal that oridonin, by directly activating HSF1, triggers chaperone-mediated degradation of BCR-ABL in Ph^+^ leukemias. This mechanism is totally different from that of imatinib, which urges us to examine whether oridonin was efficacious in imatinib-resistant Ph^+^ leukemias. We transfected the BCR-ABL-negative cell line HL60 with expression vectors of BCR-ABL (p210) in either the wild-type (WT) or the mutated form, and selected for stable clones (HL60/p210). These clones were then treated with oridonin for 6 hours, and the expression of BCR-ABL was examined by western blotting. As shown in Fig. 6a, oridonin remarkably reduced BCR-ABL and its target MYC not only in HL60/p210WT cells, but also in HL60/p210T315I and HL60/p210E255K cells that were previously reported to be resistant to imatinib^6^,^8^. In agreement with these results, oridonin exhibited potent inhibitory effects in both WT and mutated HL60/p210 clones, as shown by the reduction of cell growth (Fig. 6b) and the induction of apoptosis (Fig. 6c), whereas imatinib was less efficacious in cells with mutated BCR-ABL (Fig. 6b). Furthermore, in nude mice simultaneously injected s.c. over the right and left shoulder with HL60/p210WT and HL60/p210 T315I cells, respectively, oridonin substantially inhibited not only the growth of xenograft tumors with WT BCR-ABL but also those with imatinib-resistant T315I-BCR-ABL (Fig. 6d,e). Immunohistochemistry with an anti-c-Abl antibody revealed that oridonin decreased the signal in both WT and mutated tumors, which was not the case for imatinib (Fig. 6f).

## Discussion

**Figure 7:**
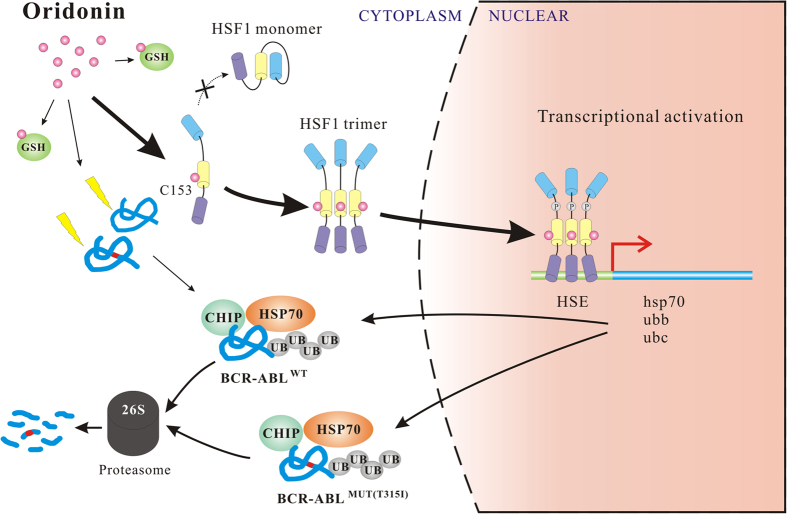
Working model for the mechanism of oridonin in leukemia.

Although it is widely acknowledged that the onset of cancer is a complex process driven by the accumulation of multiple genetic mutations^31^,^32^, the concept of “oncogene addiction” indicates a promising approach towards cancer therapy by hitting the “Achilles’ heel” within the cancer cell^33^. The BCR-ABL oncogenic protein is such an “Achilles’ heel” in Ph-positive cancers, especially in CML. In the present study, we show that the natural diterpenoid compound oridonin, which acts through posing oxidative stress in cancer cells, activates HSF1 and HSP70 and targets BCR-ABL for ubiquitin-proteasome degradation (Fig. 7). This novel mechanism of action makes oridonin efficacious against Ph^+^ leukemia cells regardless of the BCR-ABL mutation status.

Oridonin has been suggested to have the potential to react with thiol groups^30^. We demonstrated that oridonin decreases the total cellular thiol pool, which was supported by a previous report showing depletion of GSH and inhibition of components in the thioredoxin system by oridonin^14^. The lowering of the levels of these antioxidants, together with the increase in the ROS level, might result in the remarkable apoptosis that is induced by oridonin because these cytotoxic effects of oridonin can be largely reversed by the addition of DTT (functions by antagonizing thiol oxidization, Fig. 5) or NAC (functions as an ROS scavenger, Supplementary Fig. S5). From this point of view, the action of oridonin is somewhat similar to that of the oxidants as expounded in a recent review^34^.

We therefore hypothesized that one or more key target proteins that may have been protected by the cellular reducing buffer would be exposed to oridonin and thus mediate its anti-leukemia activity. Indeed, using ESI-MS and DTNB-thiol assays, we revealed that oridonin bound directly to HSF1 and reacted with the thiols in its cysteines, with especially high affinity for the C153 residue, implying that the C153 of HSF1 would be more easily exposed to oridonin even under the protection of a reducing buffer such as GSH. It has been reported that a high-concentration thiol-reducing buffer favors the formation of intramolecular disulfide covalent bonds between the C153, C373, and C378 residues of HSF1, whereas a low-concentration thiol-reducing reagent cleaves these bonds and promotes activation of HSF1^35^. Therefore, the effect of oridonin on HSF1 can be explained by the model that in the presence of oridonin, the concentration of the thiol-reducing buffer decreases, allowing for the exposure of and the binding of oridonin to cysteine 153, thus preventing the formation of intramolecular disulfide covalent bonds (Fig. 7). This creates favorable conditions for the formation of intermolecular disulfide covalent bonds and the subsequent activation of HSF1, characterized by the trimerization, phosphorylation and nuclear localization of this protein (Fig. 7). Active HSF1 transcriptionally activates not only HSP70 but also ubiquitin proteins, leading to CHIP-mediated degradation of several HSP90 client proteins with oncogenic functions, including BCR-ABL and its downstream target MYC. Considering our previous report that FBW7 mediated oridonin-induced degradation of MYC, the finding of CHIP pathway in MYC degradation suggests the contribution of both E3 ubiquitin ligases under the action of oridonin. In addition, according to our recent reports that oridonin targets a downstream pathway of BCR-ABL (i.e., MYC-miR-17/20a-BIM-S) to induce apoptosis and reverse chemoresistance in leukemia cells^16^,^17^, our current finding that oridonin targets BCR-ABL as well as MYC for degradation would better explain the efficacy of oridonin.

HSP70 is a member of the heat shock protein family that is known to promote de-novo folding of nascent proteins as well as refolding of mature misfolded proteins^36^. Recent studies have indicated a role of HSP70 in cancer initiation and progression independent of its chaperone function^37^,^38^; however, this protein seems to be a double-edged sword in cancer because it was shown that membrane-bound and extracellular HSP70 can elicit anti-tumor immune responses^39^. Moreover, as a chaperone, the role of HSP70 can be shifted from protein folding pathway to the degradation pathway in cooperation with the cochaperone CHIP as previously reported^40^,^41^,^42^, and this would be beneficial when the substrate is a so-called “Achilles’ heel”. Interestingly, another cochaperone, Hop (p60), which promotes protein stabilization, was not induced by oridonin (data not shown). Oridonin poses oxidative stress to target proteins by reacting with thiol groups in cancer cells, and at the same time enhances the expression of HSP70 and polyubiquitin genes by activating HSF1, which likely shifts the activity of HSP70 from protein folding to degradation as seen from the subsequent degradation of a group of oncogenic client proteins, including BCR-ABL and MYC. Therefore, the induced expression of HSP70 by oridonin is different from the accompanied induction of HSP70 by HSP90 inhibitors that was shown to weaken their efficacy^43^. Oridonin is different from HSP90 inhibitors because it does not inhibit the association of HSP90 with its client proteins, such as BCR-ABL and MYC (Supplementary Fig. S6a). In addition, unlike the HSP90 inhibitor 17-AAG, oridonin does not affect the phosphorylation status of HSP90 but increases the levels of p-HSP70 (Supplementary Fig. S6b). Thus, in this sense, oridonin can be considered a novel pharmaceutical HSP70 and ubiquitin inducer that kills cancer cells by triggering chaperone-mediated degradation of oncogenic proteins.

## Materials and Methods

### Cell lines and reagents

K562 and HL60 cells were from Dr. Shimei Zhuang (Sun Yat-sen University, Guangzhou, China) while SUP-B15 cells were from Dr. Yueqin Chen (Sun Yat-sen University). KU812 cells were obtained from the cell bank of Chinese Academy of Sciences (Shanghai, China). All these lines had been authenticated using short-tandem-repeat (STR) profiling in the last six months.

Oridonin (JiShi Pharmaceutical) was dissolved in DMSO (Sigma) at a concentration of 40 mM and stored at −20 °C. Imatinib mesylate (Gleevec, Novartis Pharmaceuticals) was prepared as a 10 mM stock solution in PBS. Dithiothreitol (DTT) was made as a 1 M stock solution and kept at −20 °C. The stock solution of cycloheximide (CHX, 50 mg/ml, Beyotime), MG132 (50 mM, Calbiochem), ALLN (100 mM, Calbiochem) and lactacystin (10 mM, Calbiochem) were prepared in DMSO and used at 50 μg/ml, 10 μM, 100 μM and 25 μM, respectively. The final concentrations of DMSO were kept below 0.05% in all cell cultures.

### SiRNAs and Plasmids

All chemically synthesized siRNAs were purchased from Genepharma and were transfected at a final concentration of 50 nM. The siRNA sequences are listed in Supplementary Table S1.

To construct reporter genes, the PCR products of HSP70, UBB and UBC promoter regions were incorporated into the pGL3-T vector between the KpnI and HindIII sites. The mutant plasmids were then constructed by site-directed mutagenesis using a Multipoints Mutagenesis Kit (Takara). The sequences of the primers are listed in Supplementary Table S2.

The wild-type BCR-ABL (p210)-pcDNA3 expression vector was kindly provided by Dr. Jinxuan Pan (Sun Yat-sen University). The mutant plasmids containing the E255K or T315I mutations were obtained from TaKaRa.

### Transfection and generation of stable cell lines

All transfections of plasmids and siRNAs were performed using the Neon transfection system (Life Technologies) according to the instructions of the manufacturer.

HL-60/p210^wt^, HL-60/p210^T315I^, and HL-60/p210^E255K^ cells were generated by transfection of the wild-type or mutant p210 expression vectors into HL60 cells. Stable clones were selected with 1 mg/ml G418 (Sigma). Surviving cells were subcloned by limiting dilution and resistant colonies were expanded to stable cell lines and maintained in the presence of 500 μg/ml G418.

### Cell viability and apoptosis assays

Cell viability was determined using 3-(4,5-dimethylthiazol-2-yl)-2,5-diphenyltetrazolium bromide (MTT) assays (Promega), and apoptosis was measured by flow cytometry using FITC-Annexin V/PI staining as previously described^16^.

### Quantitative RT-PCR (qPCR)

RNA was isolated from cells using Trizol reagent (Life Technologies). First-strand cDNA was synthesized using a PrimeScript™ RT reagent kit with gDNA Eraser (Takara). Realtime PCR was performed using SYBR^®^ Premix Ex Taq™ II (Takara) and the StepOne Real-Time PCR System (Applied Biosystems). GAPDH was used as an internal control. Primers for qPCR are listed in Supplementary Table S3.

### Protein arrays and immunoblotting

Cells were lysed with 1× cell lysis buffer (Cell Signaling Technology, CST). The activities of 28 receptor tyrosine kinases and 11 downstream signaling kinases were detected using the RTK Signaling Antibody Array Kit (CST, see Supplementary Table S4 for list of the kinases). The protein arrays were visualized by fluorescent staining, scanned using the Odyssey CLx Infrared Imaging System (LI-COR Biosciences) and quantified using Image Master TotalLab V2.0 (Amersham Pharmacia Biotech).

Immunoblotting was performed as previously described^16^,^44^. Band intensity was quantitated when necessary using ImageMaster Totalab software. Information about the antibodies used for immunoblotting is provided in Supplementary Table S5.

### Immunoprecipitation (IP)

IPs were carried out as previously described^7^,^16^. Briefly, Cells were lysed with immunoprecipitated lysis buffer (Beyotime) at 6 × 10^6^ cells/mL for control samples and 1.2 × 10^7^ cells/mL for oridonin-treated samples. Proteins were immunoprecipitated from equal volumes of cell lysates using anti-MYC or anti-c-abl antibody. Rabbit IgG (Millipore) was used as a control. The immune complexes were washed three times with lysis buffer and subjected to immunoblotting analysis.

For detection of c-ABL specific ubiquitination, K562 cells were pretreated with 10 μM MG-132 for 2 hours and then treated with DMSO or 20 μM oridonin for 1 hour. Equal numbers of cells were lysed and subjected to IP with Rabbit IgG or anti-c-abl antibody, flowed by immunoblotting analysis with anti-ubiquitin or anti-c-abl antibody.

### Immunofluorescence and confocal microscopy

Cells were collected and immediately fixed in 4% paraformaldehyde for 10 minutes. Fixed cells were loaded onto coverslips, permeabilized with 0.2% Triton X-100 for 10 minutes, and blocked with 5% BSA for 1 hour. Single immunostaining was performed as previously described^17^, and double-staining was performed using a sequential method. After staining the cells with DAPI (1 μg/mL) for 10 minutes, the coverslips were mounted and sealed. The stained cells were visualized using a TCS SP5 confocal microscope (Leica) and LSM 710 Duo NLO confocal microscope (Zeiss).

### Luciferase reporter assays

K562 cells (2 × 10^5^) were transfected with 400 ng wild-type or mutant HSE reporter plasmids plus 150 ng pRL-TK plasmid. Forty-eight hours later, the cells were replated in duplicate and treated with DMSO or 10 μM oridonin. Cell extracts were prepared and assayed for luciferase activity using the Dual-luciferase Reporter Assay System (Promega) 24 hours later.

### Total thiol (TSH) determination and the DTNB assay

For the determination of TSH, cell lysates were incubated with 5,5′-dithiobis-(2-nitrobenzoic acid) (DTNB, Beyotime) for 5 minutes at room temperature, and the absorbance at 410 nm was detected.

The DTNB assay was used to evaluate the oxidation of thiols in peptides by oridonin. The synthetic peptides (GL Biochem) were dissolved in appropriate buffer as instructed to make stock solutions of 5 mM, then mixed with oridonin or DMSO and incubated at 37 °C for 10 minutes. DTNB was added and the absorbance at 410 nm was measured.

### Electrospray ionization mass spectrometry (ESI-MS)

Peptides were diluted in a mixture of methanol/water (50:50, v/v) to a final concentration of 100 μM immediately before use. DMSO or oridonin was added to the diluted peptides at a 1:1 molar ratio and incubated for 10 minutes at room temperature. A four-fold volume of methanol was added to obtain a good spray. ESI-MS spectra were then obtained using a Finnigan LCQ LCQ DECA XP LC/MS Mass Spectrometer (Thermo Finnigan) or a TSQ Quantum Ultra Triple Stage Quadrupole Mass Spectrometer (Thermo Finnigan) in the Instrumental Analysis & Research Center in Sun Yat-sen University.

### *In vitro* HSF1 cross-linking

Whole-cell extracts were prepared by repeated thawing of frozen cell pellets in 1× PBS plus 0.5 mM EDTA and protease inhibitor cocktail (Roche) followed by incubation on ice for 15 minutes. Supernatants were used for HSF1 trimerization studies as previously described^45^. Briefly, EGS [ethylene glycol bis(succinimidyl succinate)] was added to the cell lysates at a final concentration of 1 mM and incubated at room temperature for 30 minutes. After quenching the reactions with excess glycine, the samples were resolved by 8% SDS-PAGE and analyzed by immunoblotting.

### Xenograft tumor models

All procedures for the experiments using mice were approved and performed in accordance with relevant guidelines and regulations by the Animal Care and Use Committee of Sun Yat-sen University. HL60/p210^WT^ and HL60/p210^T315I^ cells were resuspended at 4.5 × 10^7^ cells/mL in 2:1 non-supplemented IMDM media and Matrigel (BD Biosciences). Each 5-week-old BALB/c nude mouse was subcutaneously injected with HL60/p210^WT^ cells over the right shoulder and HL60/p210^T315I^ cells over the left shoulder. When the tumors reached a volume of 150 mm^3^, the mice were randomized into three groups (6 mice/group) and intraperitoneally administered with oridonin (15 mg/kg), imatinib (15 mg/kg) or vehicle (2% DMSO) once a day for 12 consecutive days. Tumor volumes (tumor length * width^2^ * 0.5236) and body weights were monitored daily. All mice were sacrificed one day after the final treatment. Tumors were fixed in paraformaldehyde before subjected to immunohistochemistry.

### Statistical analysis

The data are the means of three independent experiments (three replicates each). Error bars indicate the SD. Statistical differences were assessed using two-tailed unpaired Student’s t test. *P* < 0.05 was considered statistically significant.

## Additional Information

**How to cite this article**: Huang, H. *et al*. Oridonin Triggers Chaperon-mediated Proteasomal Degradation of BCR-ABL in Leukemia. *Sci. Rep.* **7**, 41525; doi: 10.1038/srep41525 (2017).

**Publisher's note:** Springer Nature remains neutral with regard to jurisdictional claims in published maps and institutional affiliations.

## Supplementary Material

Supplementary Information
