# Supplementary material for: Oridonin Triggers Chaperon-mediated Proteasomal Degradation of BCR-ABL in Leukemia
Source: Sci Rep. 2017 Jan 27;7:41525. doi: 10.1038/srep41525 (PMC5270248; doi:10.1038/srep41525)
Supplement: Supplementary Information [file srep41525-s1.docx]

**Title**

Oridonin Triggers Chaperon-mediated Proteasomal Degradation of BCR-ABL in Leukemia

**Authors Information**

Huilin Huang^1^, Hengyou Weng^1^, Bowen Dong, Panpan Zhao, Hui Zhou*, Lianghu Qu*

^1^ These authors contributed equally to this work.

* Correspondence should be addressed to Lianghu Qu or Hui Zhou, Guangzhou 510275, PR China; Tel: +86 20 84112399; Fax: +86 20 84112399; E-mails: lsszh@mail.sysu.edu.cn or lssqlh@mail.sysu.edu.cn.

All the authors are affiliated with Key Laboratory of Gene Engineering of the Ministry of Education, State Key Laboratory for Biocontrol, Sun Yat-sen University, Guangzhou 510275, China.

**Supplementary Information**

**Supplementary Figures**


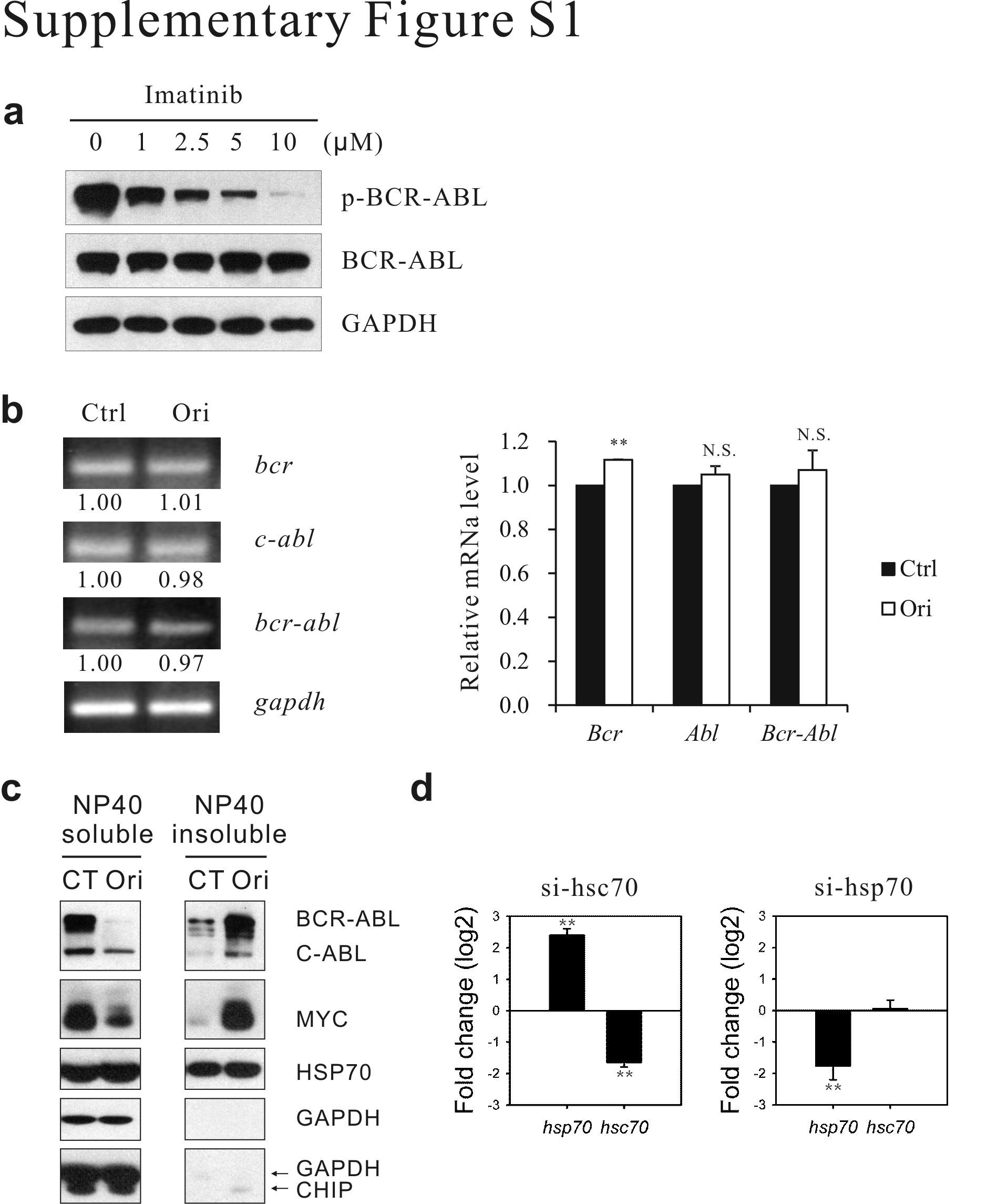


**Supplementary Figure S1** Oridonin downregulates BCR-ABL protein.

(a) Concentration-dependent inhibition of BCR-ABL kinase activity by imatinib. (b) K562 cells were treated with DMSO or 20 μM oridonin for 24h and subjected to RT-PCR (left) and qPCR (right) for the detection of bcr, c-abl and bcr-abl mRNA levels. Ctrl: DMSO; Ori: oridonin. **, P<0.01; N.S., non-significant. (c) After 24h-treatment of DMSO (CT) or oridonin (20 μM), K562 cells were lysed with NP40 on ice. The NP40 insoluble fraction was spinned down and resolved with 2% SDS. Both NP40 soluble and insoluble fractions were loaded onto SDS-PAGE gel and subjected to immunoblotting. (d) K562 cells were transfected with siRNA against hsc70 or hsp70. Cells were lysed 48h later and the mRNA levels of hsc70 and hsp70 were examined by qPCR.


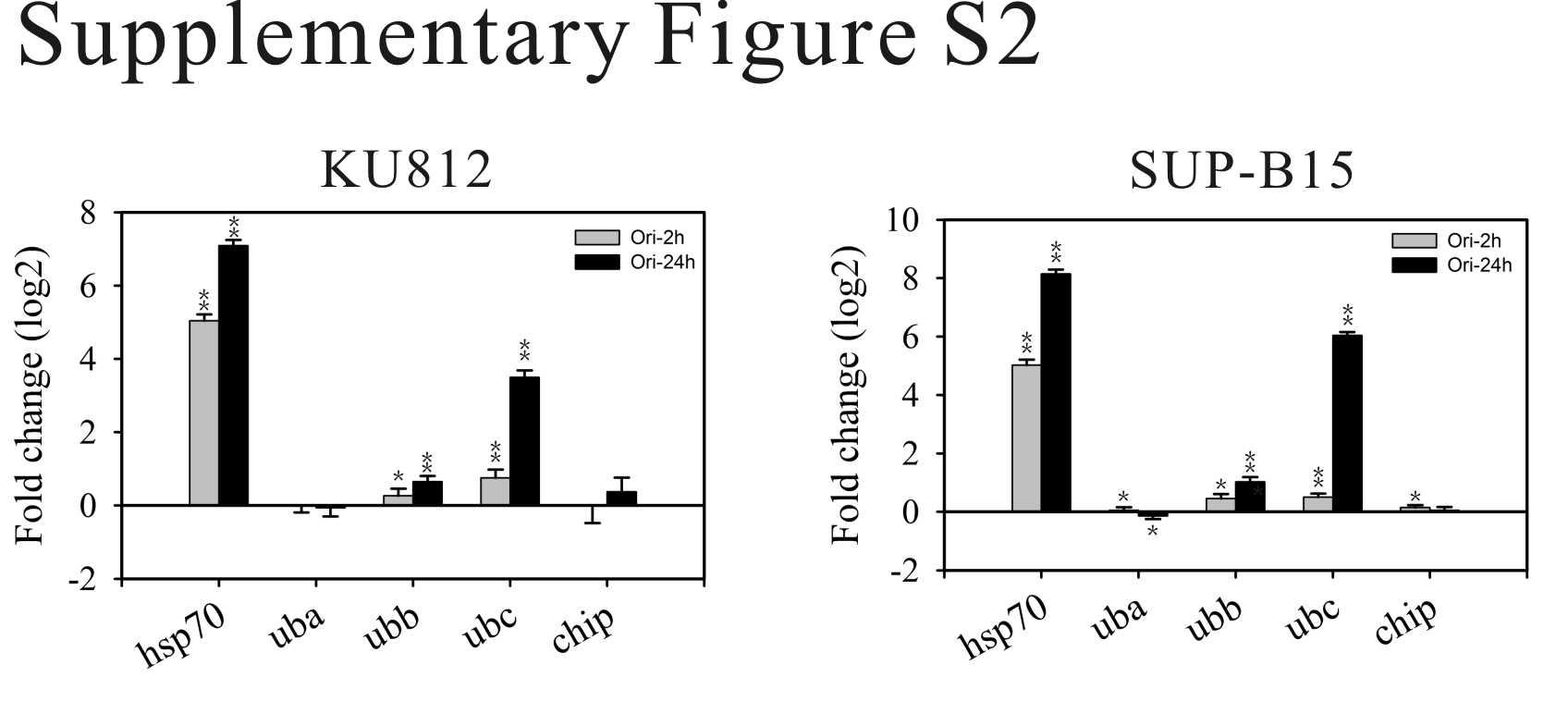


**Supplementary Figure S2** Oridonin induces degradation of BCR-ABL protein through chaperone-mediated ubiquitin-proteasome pathway.

Quantitative PCR detection of mRNA expression of genes in KU812 and SUP-B15 cells treated with oridonin (20 μM) as compared to the corresponding DMSO-treated cells. *, P <0.05; **, P<0.01.


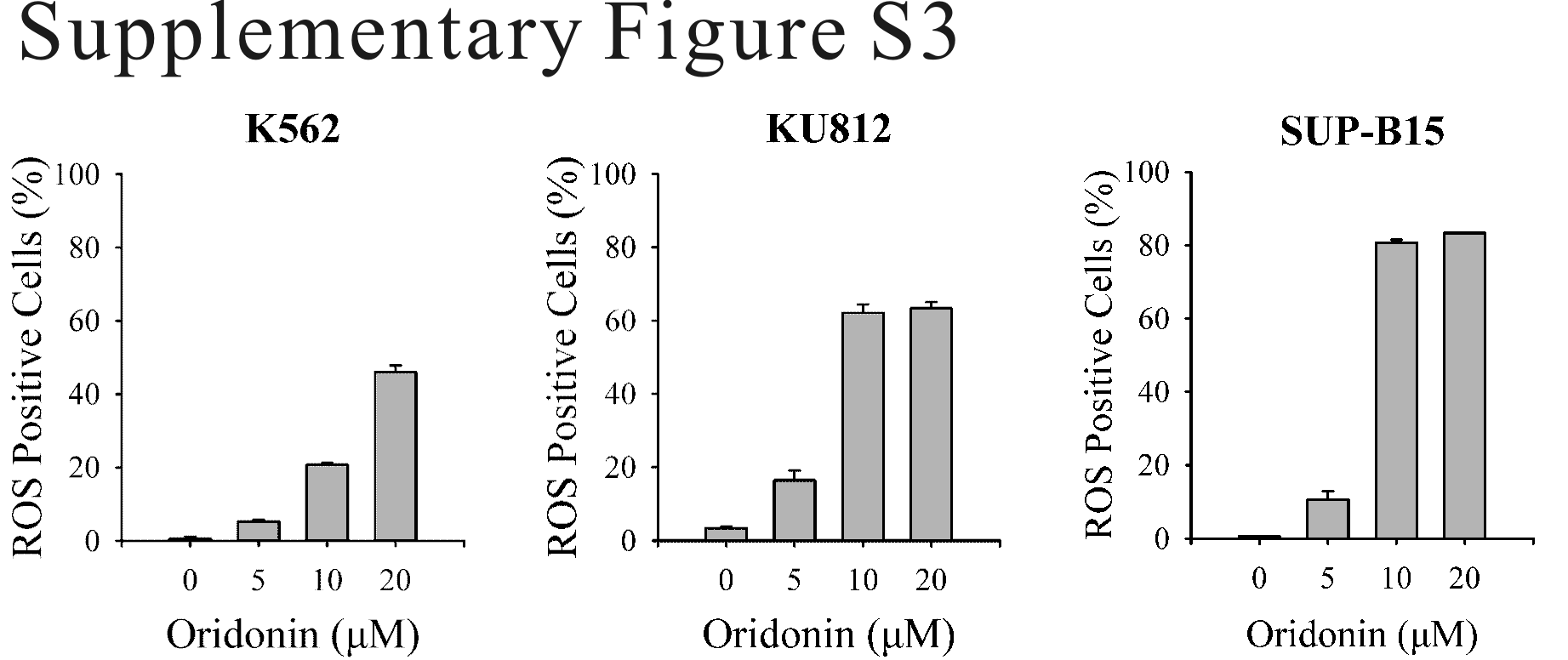


**Supplementary Figure S3** Oridonin induces ROS in Ph+ leukemia cells.

Cells were treated with oridonin for 2h as indicated and subjected to ROS assay in a flow cytometer.


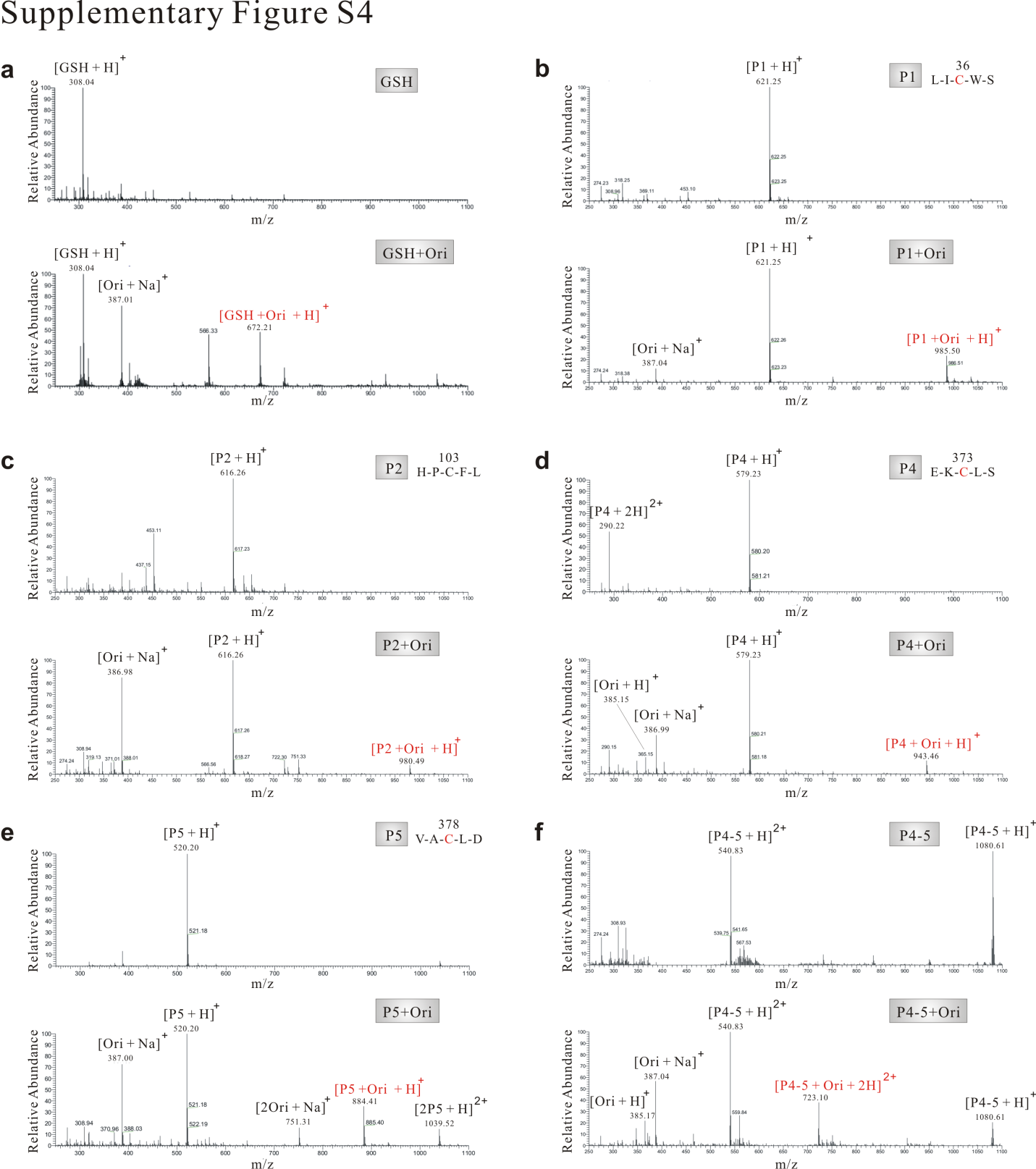


**Supplementary Figure S4**. ESI-MS showing the interaction between oridonin and the cysteine-containing peptides.

GSH (a) and peptides containing one cysteine (b-e) or two cysteines (f) of HSF1 were incubated with DMSO or oridonin at a 1:1 molar ratio at room temperature for 10 minutes and loaded for ESI-MS.


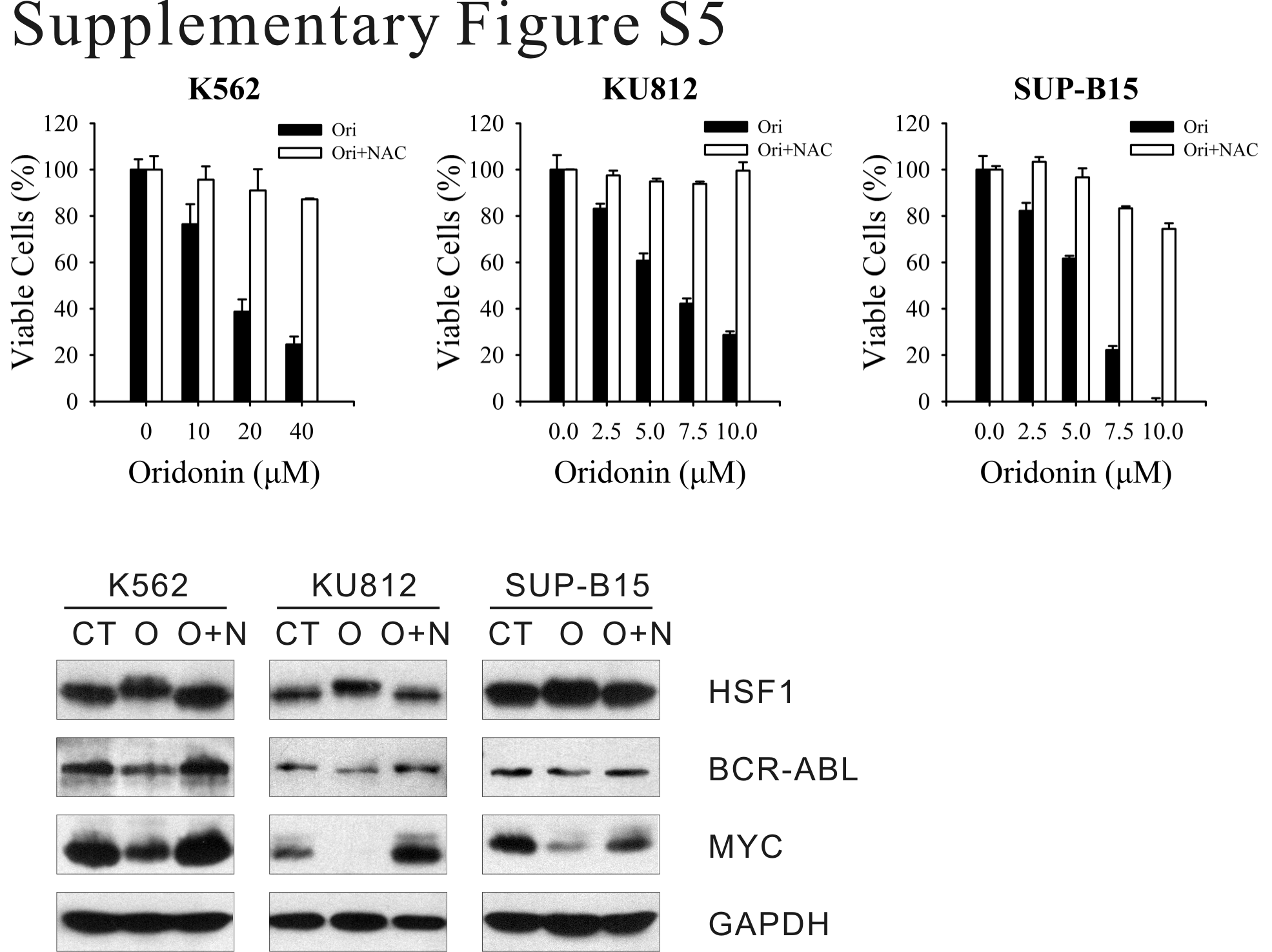


**Supplementary Figure S5**.The ROS inhibitor, NAC, prevents the oridonin-induced cytotoxic effects and degradation of BCR-ABL and MYC in Ph+ leukemia cells.

Upper, NAC abrogates the inhibition of cell growth by oridonin as assessed by MTT assays; Lower, Cells were pre-incubated with NAC (1 mM) for 2 hours and then treated with oridonin (K562: 20 μM for 2 hours; KU812 and SUP-B15: 10 μM for 4 hours) before subjected to immunoblotting.


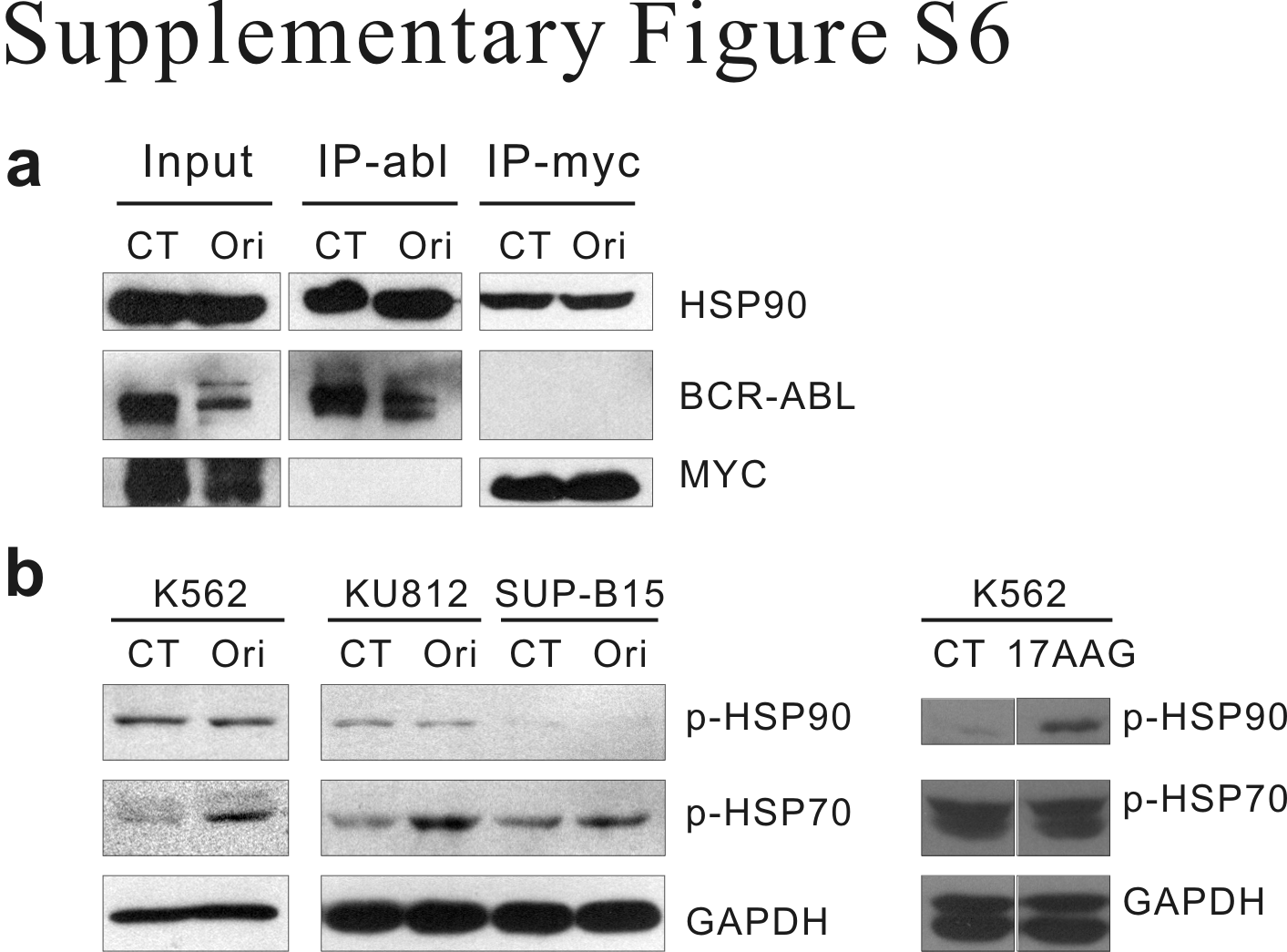


**Supplementary Figure S6**. Oridonin is not an HSP90 inhibitor.

(a) K562 cells treated with DMSO or oridonin (20 μM) for 1h were washed and lysed with IP lysis buffer and subjected to immunoprecipitation with an anti-c-Myc or anti-c-ABL antibody. The levels of HSP90 protein in these IP products were then examined by immunoblotting. (b) Oridonin increases p-HSP70 but not p-HSP90 in Ph+ leukemia cell lines, whereas 17AAG increases p-HSP90 but not p-HSP70. Cells were treated with oridonin (20 μM for K562, 10 μM for KU812 and SUP-B15) or 17AAG (5 μM for K562) for 2 hours and subjected to immunoblotting.

**Supplementary Tables**

**Supplementary Table S1** Sequences of siRNAs

| siRNA | Sequence |
| --- | --- |
| si-Hsc70 | CUGUCCUCAUCAAGCGUAA |
| si-Hsp70 | CCAAGCAGACGCAGAUCUU |
| si-HSF1-1 | CGGAUUCAGGGAAGCAGCUGGUGCA |
| si-HSF1-2 | GCUCAUUCAGUUCCUGAUCUU |
| si-NC | UUCUCCGAACGUGUCACGU |

**Supplementary Table S2** Primers for vector construction

| Primer | Sequence |
| --- | --- |
| hsp70-F | TTGGTACCAGGCGGTCAGCATCGCCAT |
| hsp70-R | TTTAAGCTTTAGCCGTTATCCGGACCGCTT |
| hsp70-mut-1 | TCCAGTGAATCCCACTAGACAGTGGAGAGTTCTGAGCAG |
| hsp70-mut-2 | AGGCGAAACCCCTGCTATAAACCCGACCTGGCAGC |
| UBB-F | TTGGTACCCAGCAACAACAGAGCCACCG |
| UBB-R | TTTAAGCTTGACACAAACAAGCTCACCCAACA |
| UBB-mut | GTGCCGGCGCTGCAAGCTAGTAACCACTGCTTTCGAGGAAGG |
| UBC-F | TTGGTACCCCTGCACTGAAATAAGGTTGGGATTC |
| UBC-R | TTTAAGCTTGCGGCCCGCGTTCCTTAGC |
| UBC-mut-1 | CCCATTGAAACCAGACTAATAACCATGCCTCCCTGTTGGC |
| UBC-mut-2 | GCAGTGTCTCCCCTGCTAAGAACTCCTGCGTTCCCCAGCTTTAG |

**Supplementary Table S3** Primers for qPCR

| Primer | Sequence |
| --- | --- |
| hsc70-F | GTGGCTTCCTTCGTTATTGG |
| hsc70-R | CGATCAACCGTTCAGTGTCC |
| hsp70-F | GAAGGACATCAGCCAGAACAAGC |
| hsp70-R | GGAACAGGTCGGAGCACAGCT |
| UBA-F | TATGCTCGCCTTCACCCTCG |
| UBA-R | GATGCCCACTCTTCAGCCAC |
| UBB-F | ATTTAGGGGCGGTTGGCTTT |
| UBB-R | ATTTTGACCTGTTAGCGGATACC |
| UBC-F | GTCCCTTCTCGGCGATTCTG |
| UBC-R | CGATCACAGCGATCCACAAA |
| CHIP-F | GAAGCGCTGGAACAGCATTGAG |
| CHIP-R | TGTCCGCCATGTACTTGTCGTG |
| BCR-ABL-F | CCGCTGACCATCAATAAGGAAGATG |
| BCR-ABL-R | TCAGACCCTGAGGCTCAAAGTCAGA |
| BCR-F | CCGCTGACCATCAATAAGGAAGATG |
| BCR-R | CACAAAATACCCAAAGGAATCCACC |
| ABL-F | GCAATGTTTTTGTGGAACATGAAGC |
| ABL-R | GAAAAGGTTGGGGTCATTTTCACTG |
| GAPDH-F | TGACCTGCCGTCTAGAAAAACC |
| GAPDH-R | GCCAAATTCGTTGTCATACCAGG |

**Supplementary Table S4** Kinases in the RTK Signaling Antibody Array

| ID | Row | Column | Tagert | Phosphorylation site | Family |
| --- | --- | --- | --- | --- | --- |
| 1 | 1 | A | Positive Control |  |  |
| 2 | 1 | B | Positive Control |  |  |
| 3 | 1 | C | FGFR1 | pan-Tyr | FGFR |
| 4 | 1 | D | FGFR1 | pan-Tyr | FGFR |
| 5 | 1 | E | TrkA/NTRK1 | pan-Tyr | NGFR |
| 6 | 1 | F | TrkA/NTRK1 | pan-Tyr | NGFR |
| 7 | 1 | G | ALK | pan-Tyr | LTK |
| 8 | 1 | H | ALK | pan-Tyr | LTK |
| 9 | 1 | I | Positive Control |  |  |
| 10 | 1 | J | EphA1 | pan-Tyr | EphR |
| 11 | 1 | K | EphA1 | pan-Tyr | EphR |
| 12 | 1 | L | EphB4 | pan-Tyr | EphR |
| 13 | 1 | M | EphB4 | pan-Tyr | EphR |
| 14 | 1 | N | Akt/PKB/Rac | Thr308 | Akt |
| 15 | 1 | O | Akt/PKB/Rac | Thr308 | Akt |
| 16 | 1 | P | IRS-1 | pan-Tyr | IRS |
| 17 | 1 | Q | IRS-1 | pan-Tyr | IRS |
| 18 | 1 | R | Positive Control |  |  |
| 19 | 2 | A | EGFR/ErbB1 | pan-Tyr | EGFR |
| 20 | 2 | B | EGFR/ErbB1 | pan-Tyr | EGFR |
| 21 | 2 | C | FGFR3 | pan-Tyr | FGFR |
| 22 | 2 | D | FGFR3 | pan-Tyr | FGFR |
| 23 | 2 | E | TrkB/NTRK2 | pan-Tyr | NGFR |
| 24 | 2 | F | TrkB/NTRK2 | pan-Tyr | NGFR |
| 25 | 2 | G | PDGFR | pan-Tyr | PDGFR |
| 26 | 2 | H | PDGFR | pan-Tyr | PDGFR |
| 27 | 2 | I | Positive Control |  |  |
| 28 | 2 | J | EphA2 | pan-Tyr | EphR |
| 29 | 2 | K | EphA2 | pan-Tyr | EphR |
| 30 | 2 | L | Tyro-3/Dtk | pan-Tyr | Axl |
| 31 | 2 | M | Tyro-3/Dtk | pan-Tyr | Axl |
| 32 | 2 | N | Akt/PKB/Rac | Ser473 | Akt |
| 33 | 2 | O | Akt/PKB/Rac | Ser473 | Akt |
| 34 | 2 | P | Zap-70 | pan-Tyr | Zap-70 |
| 35 | 2 | Q | Zap-70 | pan-Tyr | Zap-70 |
| 36 | 2 | R | Positive Control |  |  |
| 37 | 3 | A | HER2/ErbB2 | pan-Tyr | EGFR |
| 38 | 3 | B | HER2/ErbB2 | pan-Tyr | EGFR |
| 39 | 3 | C | FGFR4 | pan-Tyr | FGFR |
| 40 | 3 | D | FGFR4 | pan-Tyr | FGFR |
| 41 | 3 | E | Met/HGFR | pan-Tyr | NGFR |
| 42 | 3 | F | Met/HGFR | pan-Tyr | NGFR |
| 43 | 3 | G | c-Kit/SCFR | pan-Tyr | PDGFR |
| 44 | 3 | H | c-Kit/SCFR | pan-Tyr | PDGFR |
| 45 | 3 | I | Negative Control |  |  |
| 46 | 3 | J | EphA3 | pan-Tyr | EphR |
| 47 | 3 | K | EphA3 | pan-Tyr | EphR |
| 48 | 3 | L | Axl | pan-Tyr | Axl |
| 49 | 3 | M | Axl | pan-Tyr | Axl |
| 50 | 3 | N | p44/42 MAPK (ERK1/2) | Thr202/Thr204 | MAPK |
| 51 | 3 | O | p44/42 MAPK (ERK1/2) | Thr202/Thr204 | MAPK |
| 52 | 3 | P | Src | pan-Tyr | Src |
| 53 | 3 | Q | Src | pan-Tyr | Src |
| 54 | 3 | R | Stat3 | Tyr705 | Stat |
| 55 | 4 | A | HER3/ErbB3 | pan-Tyr | EGFR |
| 56 | 4 | B | HER3/ErbB3 | pan-Tyr | EGFR |
| 57 | 4 | C | InsR | pan-Tyr | Insulin R |
| 58 | 4 | D | InsR | pan-Tyr | Insulin R |
| 59 | 4 | E | Ron/MST1R | pan-Tyr | NGFR |
| 60 | 4 | F | Ron/MST1R | pan-Tyr | NGFR |
| 61 | 4 | G | FLT3/Flk2 | pan-Tyr | PDGFR |
| 62 | 4 | H | FLT3/Flk2 | pan-Tyr | PDGFR |
| 63 | 4 | I | Negative Control |  |  |
| 64 | 4 | J | EphB1 | pan-Tyr | EphR |
| 65 | 4 | K | EphB1 | pan-Tyr | EphR |
| 66 | 4 | L | Tie2/TEK | pan-Tyr | Tie |
| 67 | 4 | M | Tie2/TEK | pan-Tyr | Tie |
| 68 | 4 | N | S6 Ribosomal Protein | Ser235/236 | RSK |
| 69 | 4 | O | S6 Ribosomal Protein | Ser235/236 | RSK |
| 70 | 4 | P | Lck | pan-Tyr | Src |
| 71 | 4 | Q | Lck | pan-Tyr | Src |
| 72 | 4 | R | Stat3 | Tyr705 | Stat |
| 73 | 5 | A | Positive Control |  |  |
| 74 | 5 | B | Positive Control |  |  |
| 75 | 5 | C | IGF-IR | pan-Tyr | Insulin R |
| 76 | 5 | D | IGF-IR | pan-Tyr | Insulin R |
| 77 | 5 | E | Ret | pan-Tyr | Ret |
| 78 | 5 | F | Ret | pan-Tyr | Ret |
| 79 | 5 | G | M-CSFR/CSF-1R | pan-Tyr | PDGFR |
| 80 | 5 | H | M-CSFR/CSF-1R | pan-Tyr | PDGFR |
| 81 | 5 | I | Positive Control |  |  |
| 82 | 5 | J | EphB3 | pan-Tyr | EphR |
| 83 | 5 | K | EphB3 | pan-Tyr | EphR |
| 84 | 5 | L | VEGFR2/KDR | pan-Tyr | VEGFR |
| 85 | 5 | M | VEGFR2/KDR | pan-Tyr | VEGFR |
| 86 | 5 | N | c-Abl | pan-Tyr | Abl |
| 87 | 5 | O | c-Abl | pan-Tyr | Abl |
| 88 | 5 | P | Stat1 | Tyr701 | Stat |
| 89 | 5 | Q | Stat1 | Tyr701 | Stat |
| 90 | 5 | R | Positive Control |  |  |

**Supplementary Table S5** Information of antibodies

| Antibody | Source |
| --- | --- |
| BCR-ABL pathway (p-c-Abl, p-CRKL, p-STAT5, RABII) | Cell Signaling Technology (CST) |
| c-Abl | Cell Signaling Technology (CST) |
| BCR | Cell Signaling Technology (CST) |
| GAPDH | Cell Signaling Technology (CST) |
| MYC | Cell Signaling Technology (CST) |
| HSF1 | Cell Signaling Technology (CST) |
| HSP90α/β | Santa Cruz |
| HSP70 | Santa Cruz |
| CHIP | Cell Signaling Technology (CST) |
| SRC | Cell Signaling Technology (CST) |
| HIF1A | Cell Signaling Technology (CST) |
| AKT | Cell Signaling Technology (CST) |
| RAF | Cell Signaling Technology (CST) |
| UB | ENZO |
| p-HSF1(Ser230) | Santa Cruz |
| p-HSF1(Thr142) | Sigma |
| p-HSP70 (Tyr525) | Cell Signaling Technology (CST) |
| p-HSP90 (Ser254) | Cell Signaling Technology (CST) |
